# All Single-Mode-Fiber Supercontinuum Source Setup for Monitoring of Multiple Gases Applications

**DOI:** 10.3390/s20113239

**Published:** 2020-06-06

**Authors:** Javier A. Martin-Vela, Eloisa Gallegos-Arellano, Juan M. Sierra-Hernández, Julián M. Estudillo-Ayala, Daniel Jauregui-Vázquez, Maria S. Avila-Garcia, Humberto Ramírez-Gasca, Roberto Rojas-Laguna

**Affiliations:** 1 Departamento de Ingenieria Electronica, Division de Ingenierias, Universidad de Guanajuato, Carretera Salamanca-Valle de Santiago km 3.5 + 1.8, Comunidad de Palo Blanco, Salamanca Gto. C.P. 36885, Mexico; ja.martinvela@ugto.mx (J.A.M.-V.); jm.sierrahernandez@ugto.mx (J.M.S.-H.); julian@ugto.mx (J.M.E.-A.); jaureguid@ugto.mx (D.J.-V.); rlaguna@ugto.mx (R.R.-L.); 2 Departamento de Mecatronica Universidad Tecnologica de Salamanca, Av. Universidad #200, Col. Ciudad Bajio, Salamanca Gto. C.P. 36766, Mexico; hramirez@utsalamanca.edu.mx; 3 Departamento de Estudios Multidisciplinarios, Division de Ingenierias, Universidad de Guanajuato, Av. Universidad s/n, Col. Yacatitas, Yuriria Gto. C.P. 38940, Mexico; susana.avila@ugto.mx

**Keywords:** absorption spectroscopy, supercontinuum source, fiber optics, fiber optic measurements, broadband, gas sensing, real-time monitoring, multiple gases

## Abstract

In this paper, a gas sensing system based on a conventional absorption technique using a single-mode-fiber supercontinuum source (SMF-SC) is presented. The SC source was implemented by channeling pulses from a microchip laser into a one kilometer long single-mode fiber (SMF), obtaining a flat high-spectrum with a bandwidth of up to 350 nm in the region from 1350 to 1700 nm, and high stability in power and wavelength. The supercontinuum radiation was used for simultaneously sensing water vapor and acetylene gas in the regions from 1350 to 1420 nm and 1510 to 1540 nm, respectively. The experimental results show that the absorption peaks of acetylene have a maximum depth of approximately 30 dB and contain about 60 strong lines in the *R* and *P* branches, demonstrating a high sensitivity of the sensing setup to acetylene. Finally, to verify the experimental results, the experimental spectra are compared to simulations obtained from the Hitran database. This shows that the implemented system can be used to develop sensors for applications in broadband absorption spectroscopy and as a low-cost absorption spectrophotometer of multiple gases.

## 1. Introduction

In recent years, various types of optical setups have been proposed for sensing and monitoring different gases, such as H_2_O, H_2_S, CO_2_, C_2_H_2_, CH_4_, and He, by means of absorbing radiation in the near infrared range [1,2,3,4,5,6,7,8]. Many of these setups are based on a spectroscopic absorption principle since atoms absorb light at specific wavelengths [9]. There are different techniques that are based on this principle and are used for gas monitoring and sensing such as: Tunable Diode-Laser Absorption Spectroscopy (TDLAS) [10,11,12,13,14,15,16,17,18], Tunable Laser Absorption Spectroscopy (TLAS) [19,20,21], Cavity Ring–Down Spectroscopy (CRDS) [19,22,23], Cavity Enhanced Absorption Spectroscopy (CEAS) [24], Fiber Laser Intracavity Absorption Spectroscopy (FLICAS) [25,26], gas cell with coreless fiber optic [27,28], or measurement of the incident and transmitted intensity of light travelling through a medium [29,30].

Some of these techniques use laser diodes, lasers, or tunable lasers for their high output power, narrow wavelength, and high resolution, however the poor stability of these parameters and the narrow measurable bandwidth limit the applicability of devices based on these techniques since they are tuned to detect and measure only certain absorption peaks of the molecule. Therefore, broad spectrum sources, such as those reported using Amplified Spontaneous Emission (ASE) sources [25,26,31,32,33], Super-luminescent Diodes (SLD) [29,30,34,35], Xenon arc lamps [36,37], and supercontinuum (SC) sources [34,35,38,39,40,41,42,43,44,45,46,47,48,49], are alternative sources that can be used to detect and measure the concentration of a pure gas or mixtures of gases. SC sources are built using a fiber laser pump and fiber optic [46] and are particularly useful given their wide bandwidth and output power when compared to ASE and SLD sources [34]. In Reference [38], the authors designed a SC source by connecting in series a femtosecond fiber laser emitting at 1560 nm, a non-linear fiber, a dispersive fiber, and a tunable spectral bandpass filter, and detected the absorption bands of CO_2_, C_2_H_2_, C_2_H_6_O, and H_2_O. Genty et al. [40] proposed an array of micro-structured fibers combining a SC source and a gas cell in a single micro-structured fiber. The SC source was obtained by launching nanosecond pulses from a compact, Q-switched Nd:YAG laser into a micro-structured fiber filled with acetylene.

A SC source was also obtained by launching 5 ps laser pulses, centered around 1060 nm, into 10 m of conventional single-mode fiber (Corning SMF28, Charlotte, NC, USA). The pump laser was an Ytterbium fiber laser (Fianium, FemtoPower-1060, NKT Photonics Inc., Freehold, NJ, USA) featuring a variable repetition rate [44,45,46,47]. In Reference [44], a CH_4_ sensor used a SC source and a multi pass-cell technique, while in Reference [45] the collimated SC beam was passed through 2.8 m of air to measure absorption from the H_2_O molecule. In Reference [46], the study of lines between 1500 and 1550 nm of high-temperature H_2_O in a laminar flame was reported using a SC source, as well as the CEAS technique using pure CO_2_ lines to validate the ringdown measurement. In Reference [41], a SC source and the CEAS technique for detecting O_2_ and C_2_H_2_ was reported. The SC source was obtained by pumping ~20 m of a highly nonlinear photonic crystal fiber (SC-5.0-1040, Crystal Fiber, NKT Photonics Inc., Morganville, NJ, USA) with 1.064 μm light from a Q-switched laser producing 10 ns pulse at a repetition rate of 30 kHz. In Reference [34], the authors used a SC source and the CEAS technique for detection of CO_2_ and CH_4_. The SC source was obtained by injecting 700 ps pulses from an ultra-compact fiber laser (Keopsys Kult, Keopsys, Lannion, France) operating at 1.5 μm into the anomalous dispersion regime of a standard single-mode fiber (Ocean UltraWave SLA, Furukawa, Tokio, Japan). In Reference [47], a SC emission was used to acquire high-resolution broadband absorption spectra of H_2_O, C_2_H_2_, and C_2_H_4_. Brown et al. [42] reported an experimental setup for measuring indoor and outdoor path absorption of water vapor concentrations using Differential Absorption Spectroscopy (DAS) and Spectral Pattern Recognition Differential Absorption Lidar (SPR-DIAL) techniques and a SC source. The SC was implemented by coupling sub-nanosecond laser pulses from a passively Q-switched microchip laser (JDSU NP-10620-100, JDS Uniphase corporation, San Jose, CA, USA, wavelength at 1064 nm, average power ~70 mW) into 18 m of photonic crystal fiber (Blaze Photonics SC-5.0-1040, Thorlabs Inc., Newton, NJ, USA). Recently, a supercontinuum source was used to measure the concentration of light hydrocarbon species that are found in fuel and energy applications, such as methane, acetylene, ethylene, and their mixtures [49].

In this work, a gas sensing system based on a conventional absorption technique and employing a single mode fiber supercontinuum source (SMF-SC) is presented. The SC source was implemented using pulses from a microchip laser and one kilometer of standard single-mode fiber (SMF), obtaining a flat high spectrum with a bandwidth of up to 350 nm in the region from 1350 to 1700 nm. High sensibility for real time acetylene and water vapor gas detection is achieved using such SC source and a cm-long conventional cell. The results are compared with those obtained using an EDFA source, a super luminescent diode, and the Hitran database.

## 2. Experimental Setup and Principal Operation of the System

### 2.1. Experimental Setup

**Figure 1:**
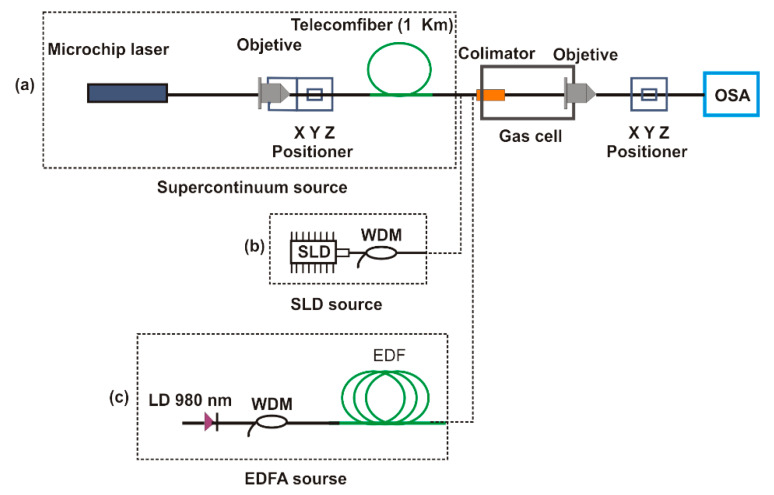
Experimental setup (**a**) Light from the supercontinuum (SC), (**b**) light from the Super-luminescent Diodes (SLD), (**c**) light from erbium-doped fiber amplified (EDFA). All are coupled to a fiber optic collimator.

**Figure 2:**
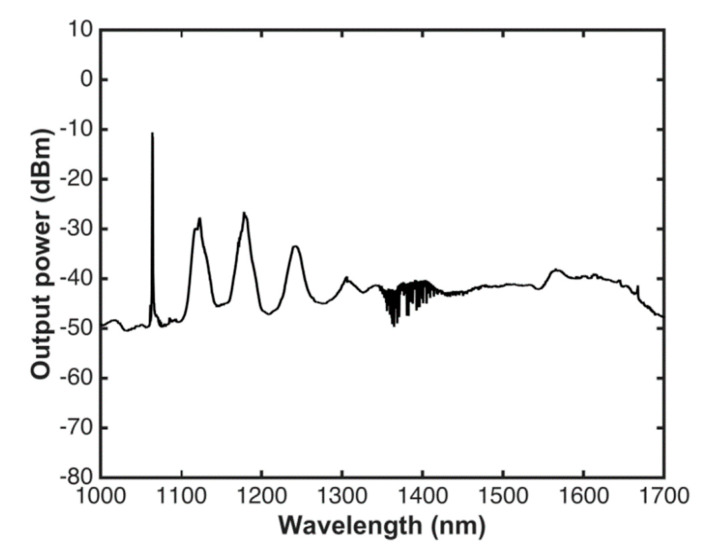
Spectral profile of the supercontinuum radiation.

In our experiment, a supercontinuum source (SCS) was used as a broadband source (see Figure 1a). Here, the SCS was implemented by pumping 1 km of a SMF on a spool with a microchip laser. The laser emits pulses with a duration of ~700 ps, an energy of 6 µJ, a repetition rate of 8.6 KHz, and a central wavelength of 1064 nm. An optical microscope objective with a magnification of 16×, and a XYZ positioner stage were used to launch the pump power from the microchip into the spool. The output light of the spool was coupled to a gas cell with a path length of 18.5 cm, reaching another microscope objective. Finally, the transmitted light was connected on an optical spectrum analyzer (OSA) with a resolution of 0.02 nm via a multimode fiber. With an input power of about 40 mW in the fiber spool, the spectrum of the pump pulse shifts to both shorter and longer wavelengths, as it is shown in Figure 2. This broadband emission is due to nonlinear effects such as modulation instability, four waves mixing, and self and cross phase modulation. For this paper, we implemented the SC source proposed in Reference [50] and in References [51,52].

The supercontinuum source presented in this work was compared to two other broad-spectrum sources: a super luminescent diode (SLD: QSMD-1550-I, Qphotonics, Ann Arbor, MI, USA) that emitted from 1400 to 1600 nm (Figure 1b) and an erbium-doped fiber amplified (EDFA) source (Figure 1c).

The EDFA source was obtained by pumping 3.5 m of erbium-doped fiber (Thorlabs, model M12-980–125, Newton, NJ, USA) with a 976 nm laser diode (Thorlabs, model BL976-PAG500, Newton, NJ, USA), obtaining a broadband spectrum from 1500 to 1600 nm.

Figure 2 shows the output spectrum of the SC source with a power of about 40 mW in the spectral region of interest, stretching from 1300 to 1700 nm for near-IR (Infrared) absorption spectroscopy of a large number of molecules, such as H_2_O, C_2_H_2_, CO, CO_2_, N_2_O, and H_2_S, among others. The absorption lines observed in the region stretching from 1340 to 1450 correspond to rovibrational overtone transitions of water vapor present in the system. Some studies show these bands of H_2_O as an example of the broadband capability of an SC-based sensor [45,47]. On the other hand, as it can be observed in Figure 2, the spectral response shape presents a high-intensity relative noise related to the pump emitting in the picosecond regime; as a result, the spectral broadening is generated mainly by a modulation instability (MI) phenomenon [53]. The MI is produced by breaking up of the pulse in many fundamental solitons, when they collide and re-combine in a chaotic manner. For this reason, the MI is a noisy process [54,55].

**Figure 3:**
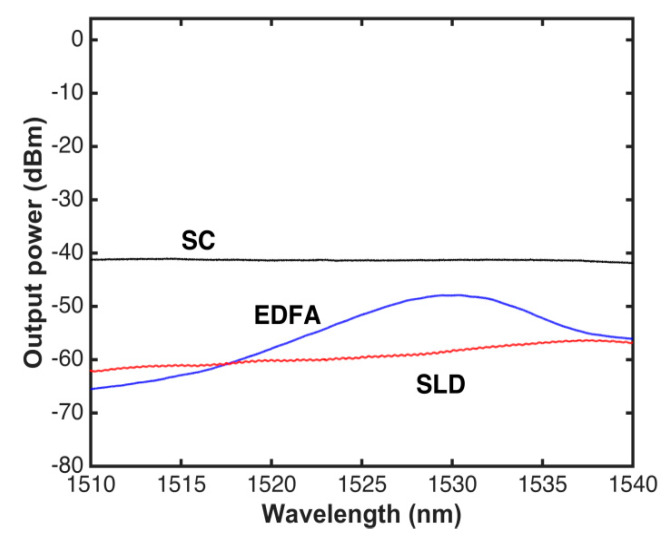
Comparison of supercontinuum (SC), super-luminescent (SLD), and fiber amplification doped with erbium (EDFA).

A direct comparison between the SLD, EDFA, and SC spectra in the wavelength band from 1510 to 1540 nm, where acetylene has strong absorption peaks, is shown in Figure 3. This figure illustrates the advantage of using the SC source for broadband spectroscopy with a power-bandwidth output wider than that of the SLD and EDFA; furthermore, the SC spectra is flat in this region. In Reference [34], it has been demonstrated that the wider the output power-bandwidth of the source, the higher the sensibility.

**Figure 4:**
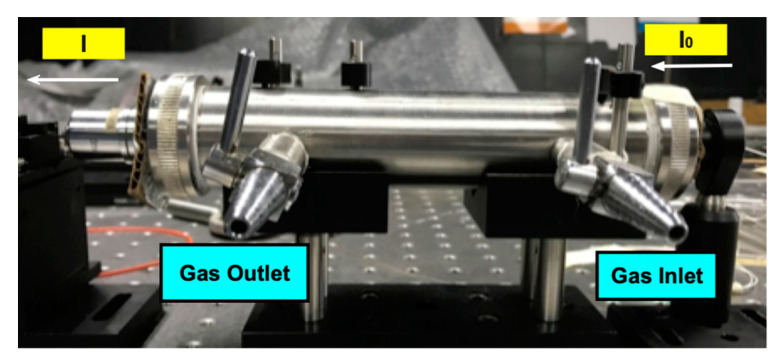
Gas cell.

The system comprises a gas cell constructed from a stainless-steel tube of 21.5 cm in length and 2.54 cm in diameter. In addition, the cell has two ports to introduce and evacuate the gas, as shown in Figure 4. Instead of windows at its ends, like a conventional cell, this one has at one end an optical fiber collimator to transmit the light coming from the source through the cell, and at the other end, a target that receives the signal and transmits it to the OSA by means of a multimode fiber (see Figure 1). It is important to note that the system must be adjusted to obtain the highest power and sensitivity.

### 2.2. Operation of the System

The system is based on the principle of absorption spectroscopy: the total transmission of light through a gas path length is described by the Beer-Lambert-Bouguer law given by [47,56]:

(1) $$T\left(\lambda ,N\right)=\frac{I\left(\lambda ,N\right)}{I_{0}\left(\lambda \right)}=e^{-LN\sigma \left(\lambda \right)}$$

where $I_{0}\left(\lambda \right)$ is the light incident intensity, I(λ,N) is the light transmitted intensity of broadband source through a gas cell before and after introducing the target gas (acetylene) into the gas cell respectively, L is the gas cell path length (cm), σ(λ) is the absorption cross-section (cm^2^/molecule), and N is the number density (molecule/cm^3^), which can be calculated as [57]:

(2) $$N=qP/\left(k_{b}T\right)$$

where *T* and *P* are the temperature and pression of the system respectively, *q* is the mole fraction, and $k_{b}$ is the Boltzmann constant. In this study, the absorption cross-section is estimated by considering the given experimental conditions and the Hitran database [58,59,60].

**Figure 5:**
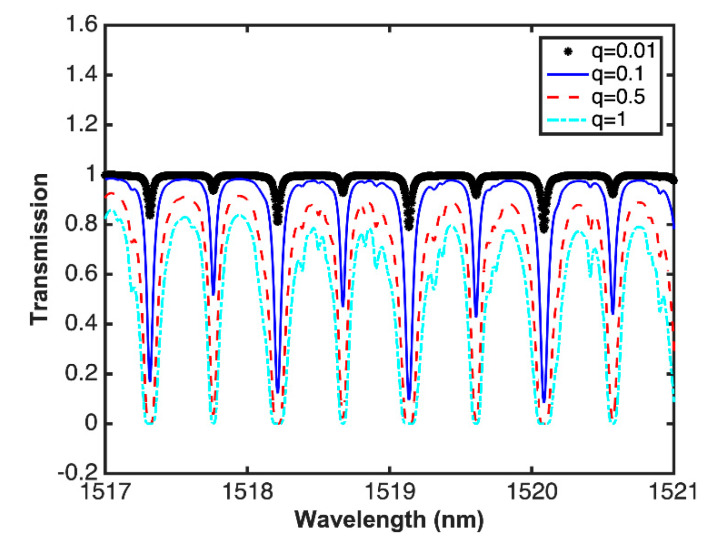
Simulations of the acetylene transmission power at different concentrations.

The simulations of the absorption spectra were carried out using Equations (1) and (2). The gas cell path length is 18.5 cm and the number density (concentration) of the target gas (in our case acetylene) is varied. Figure 5 shows simulation results of the transmission of acetylene, with *P* = 1 atm and *T* = 296 K, where it can be seen that as the gas concentration increases, a widening in the absorption line occurs, and an increase in amplitude of the absorption line is also seen.

## 3. Results and Discussion

**Figure 6:**
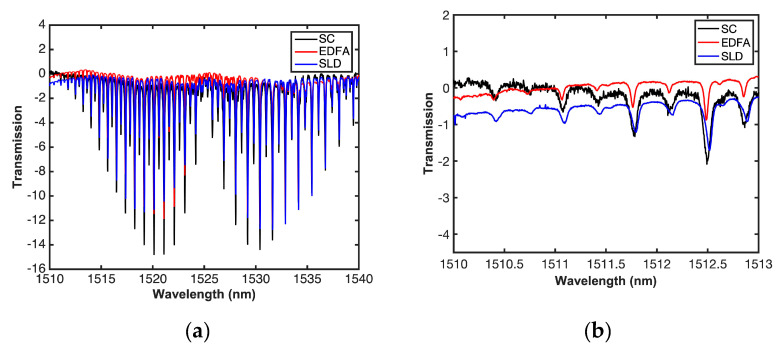
(**a**) Gas transmission with SC, SLD, EDFA, and (**b**) magnified view of 1510–1513 nm region.

Once each one of the sources was inserted, and the experimental set-up aligned, the cell was filled with 99.5% pure acetylene and measurements were made at atmospheric pressure and at room temperature. Figure 6 shows a comparison of the measurements where a supercontinuum source with a higher power-bandwidth output can provide a greater amplitude in the absorption line, as it can be seen in the R branch of the spectrum, and consequently can increase the sensitivity of the system, as demonstrated in Reference [34]. This is also shown in Figure 6b, where a magnified view of lines 29, 31, and 33, with limited absorption is shown. Furthermore, measurements with SC source show a deeper dip and better-defined absorption lines.

**Figure 7:**
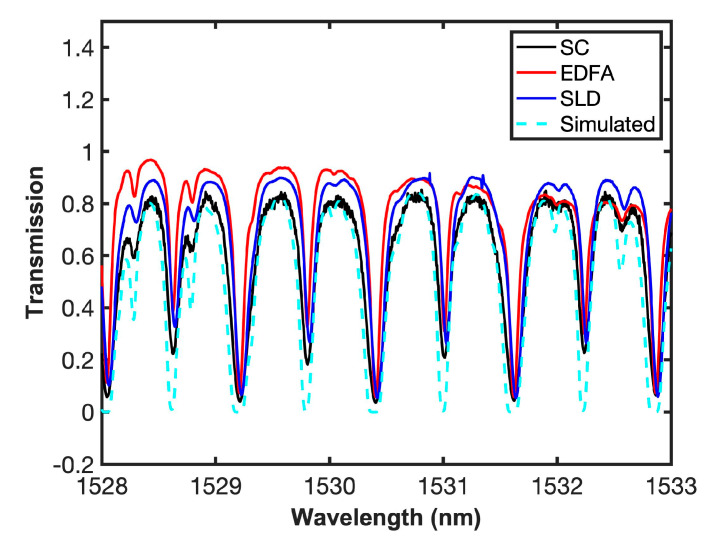
Measured and simulated gas transmission with SC, SLD, and EDFA.

Figure 7 shows a comparison of the measurements of Gas transmission with SC, SLD, EDFA, and the simulation using the parameters provided by the Hitran database, where it can be observed that the absorption lines of the simulated data are deeper, while the depth of the experimental data are limited by the sensitivity of the system.

**Figure 8:**
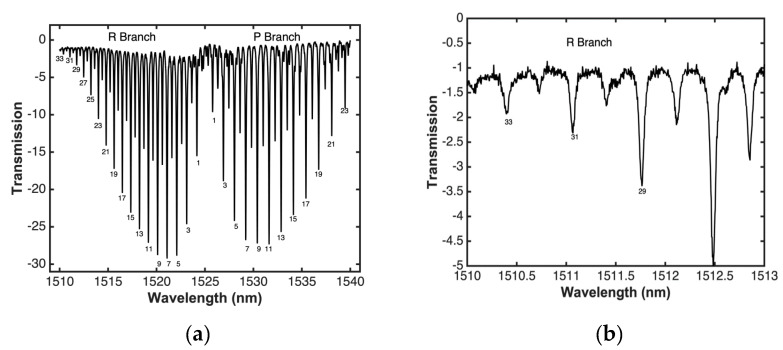
(**a**) Transmission of acetylene, (**b**) magnified view of lines 29, 31, and 33.

After a subsequent alignment, a greater sensitivity was obtained. Figure 8a shows that the absorption peaks have a maximum depth of approximately 30 dB and contain about 60 strong lines in the *R* and *P* branches of the 1510 to 1540 nm wavelength band. Compared to the measurements shown in Figure 6a, with this new alignment, a greater sensitivity can be obtained, and it is possible to have a wider detection range of ^12^C_2_H_2_ acetylene (v1 + v3 rotational–vibrational combination band). Figure 8b shows lines 29–33 of the R branch clearly, which have little intensity or depth and are not commonly detected or monitored in the reported investigations [24,27,39,40], and much less using a supercontinuum source [1,39,47]. When comparing Figure 6b and Figure 8b, it can be observed that the sensibility increases when introducing higher power into the system. However, as it can be observed in Figure 8b, the spectral response shape shows some noise due to the modulation instability phenomenon. As it was mentioned previously, the MI is a noisy process induced when using a pump laser in picosecond regime [54,55]. Although this noise can be reduced by changing both the laser pumping and the type of fiber optic, this will increase the cost of the experimental setup.

**Figure 9:**
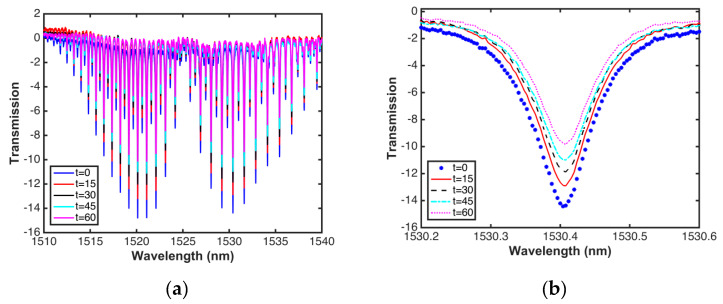
(**a**) Real-time gas monitoring with the OSA, (**b**) magnified view of 1530.2–1530.6 nm region.

To check the stability and measurement of the system in time, the monitoring of the gas concentration was performed by opening one of the valves of the cell to escape some gas. Figure 9 shows the real-time monitoring of the gas using a SCS, in 15 min time intervals, where it can be observed that as time goes by, the widening in the absorption line as well as its amplitude decrease. According to the simulations performed, the system provides a change in the concentration of gas in the cell.

**Figure 10:**
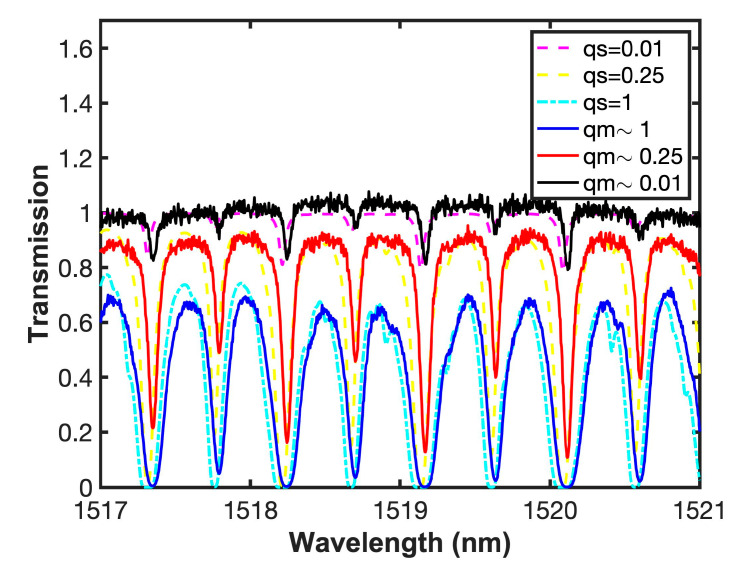
Measured (qm) and simulated (qs) gas transmission at different concentrations.

**Figure 11:**
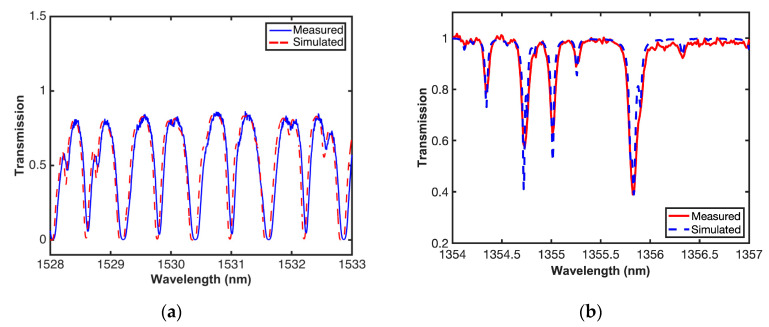
Simulated and simultaneously measured gas transmission of (**a**) acetylene and (**b**) water vapor.

Measurements were carried out by changing the concentration (molar fraction) of the gas inside the cell. Figure 10 shows a comparison between acetylene transmission in the experimental system (qm) using an OSA and the simulations (qs) using the parameters provided by the Hitran database, when *q* = 1, *q* = 0.25, and *q* = 0.1. As it can be seen, as the concentration increases, there is a widening in the absorption line, and it can also be observed that the behavior is consistent with the simulation of the system when varying the concentration. For better illustration purposes, Figure 11a shows a single comparison when *q* = 1 in the *P* Branch. It can be observed that the high resolution of the proposed system provides a clear identification of spectral lines in the *P* and *R* branches of acetylene ^12^C_2_H_2_. Furthermore, the system provides a wide wavelength coverage allowing to visualize complete spectral bands of one or more gases. Figure 11b shows both experimental and simulation results in the region between 1354 and 1357 nm, where the water vapor has an absorption band. However, as it can be observed in Figure 10 and Figure 11, the spectral response shape shows some discrepancies between transmission in the experimental system and the simulations, due to the broadening effect [61,62,63].

The proposed system does not show insertion losses since the whole arrangement is made of single-mode fiber which also makes it cheaper to manufacture compared to those made with photonic crystal fiber, which cannot be spliced to conventional fibers. Another advantage of the system is that it provides a broad wavelength coverage that allows to simultaneously visualize complete spectral bands of one or more gases, and due to its high resolution, allows the clear identification of the spectral lines of the gases. Moreover, it has high power and wavelength stability. In this work, the ^12^C_2_H_2_ acetylene bands in the *R* and *P* branches between 1510 and 1540 as well as the water vapor bands between 1354 and 1410 nm were visualized. The high sensitivity of the system with a path length of 18.5 cm using a manufactured cell was experimentally demonstrated, and it was shown that the system needs to be aligned to obtain the highest power and sensitivity. In this system, it was not necessary to lengthen the path length to increase sensitivity as it has been reported in other works. Finally, the experimental results were compared by running simulations with the data provided by the Hitran database.

## 4. Conclusions

A gas sensing system based on a conventional absorption technique by using an all single mode fiber supercontinuum source (SMF-SC) was used to simultaneously measure acetylene and water vapor. The supercontinuum source was implemented using pulses from a microchip laser and one kilometer of standard single-mode fiber (SMF), obtaining a flat, high spectrum with a bandwidth of up to 350 nm in the region from 1350 to 1700 nm, and high stability in power and wavelength. The implemented system can be used for different applications of broadband absorption spectroscopic, such as chemical analysis and simultaneous real-time monitoring of multiple gases. It can also be used to develop sensors for multiple gases or a low-cost absorption spectrophotometer capable of detecting the absorption peaks of multiple gases.
